# Retinal sensitivity and photoreceptor arrangement changes secondary to congenital simple hamartoma of retinal pigment epithelium

**DOI:** 10.1186/s40942-018-0154-7

**Published:** 2019-01-15

**Authors:** M. W. Rodrigues, D. B. Cavallini, C. Dalloul, C. L. Shields, R. Jorge

**Affiliations:** 1 Department of Ophthalmology, Ribeirão Preto School of Medicine, University of São Paulo, 3900, Bandeirantes Avenue, Ribeirão Prêto, SP 14049-900 Brazil; 2 Department of Ophthalmology, São José do Rio Preto School of Medicine, São José do Rio Preto, Brazil; 3 Department of Ophthalmology, D’Olhos, São José do Rio Preto, Brazil; 4 Ocular Oncology Service, Wills Eye Hospital, Philadelphia, USA

**Keywords:** RPE, Tumor, Hamartoma, Adaptive optics, OCT-A, Multimodal imaging

## Abstract

**Background:**

The congenital simple hamartoma of the retinal pigment epithelium is a benign lesion and previous observations with noninvasive imaging have detected potential photoreceptor abnormalities and retinal function interplay.

**Case presentation:**

A 35-year-old woman was found to have an asymptomatic, solitary, circumscribed, pigmented lesion in her left eye. The patient underwent ophthalmic examination including multimodal evaluation with fluorescein angiography, near-infrared reflectance scanning laser ophthalmoscopy, blue autofluorescence, enhanced-depth imaging spectralis B-scan optical coherence tomography (EDI-SBOCT), en face OCT angiography (OCT-A) and microperimetry plus adaptive optics imaging. Ophthalmoscopic examination revealed a juxtafoveolar pigmented lesion with feeding retinal arteriole, consistent with congenital simple hamartoma of RPE. There was no macular edema, exudation, hemorrhage, traction or subretinal fluid. Multimodal imaging of the mass using fluorescein angiography revealed intra-lesion late staining, near-infrared reflectance imaging demonstrated intrinsic hyperreflectivity, short-wavelength autofluorescence and red-free filter photography revealed blocked signal, and SBOCT showed abrupt shadowing. On OCT-A, an exclusive ring-shaped vascular circuit with increased foveal avascular zone was noted. Adaptive optics revealed cell density arrangement and retinal sensitivity correlations on microperimetry.

**Conclusion:**

These findings suggest that this hamartomatous lesion might cause specific cellular changes that impact retinal sensitivity response and potentially result from vasculature malnourishment to the outer retinal layers.

## Introduction

Congenital simple hamartoma of the retinal pigment epithelium (CSHRPE) is a rare benign pigmented lesion. Few reports have been published in the literature and most authorities presume this to be a congenital disorder [1]. This tumor is considered in the spectrum of RPE tumors based on clinical evaluation, and Gass et al. described the full thickness involvement and inner retinal surface permeation in an “umbrella” fashion [2, 3]. The largest case series has been reported by Shields et al. [1] and included clinical characteristics of five cases that demonstrated the presence of feeder vessel in 100% of cases [1]. However, most reports have failed to demonstrate any histopathologic correlation as these tumors are often observed without surgical removal. Additionally, the functional impact of this tumor has not yet been explored.

Most current multimodal imaging techniques are insufficient for evaluation of CSHRPE partly due to the presence of heavy pigmentation within the mass. The SBOCT shadowing effect is a direct result of heavy pigmentation and has failed to demonstrate the internal structure of this tumor [4, 5]. Designing a new imaging strategy that could help address this unresolved question has been challenging.

Recently, Arjmand et al. [6] and Zola et al. [7] reported a case imaged with optical coherence tomography angiography (OCT-A) for noninvasive assessment of the intrinsic retinal microvasculature. Similar to them, we explored OCT-A of this mass and found better microvasculature detail than with fluorescein angiography (FA). However, in contrast to their reports, we noted a radial microvascular arrangement underneath the pigmented mass, rather than a microvascular tangle.

Herein, we assess CSHRPE with multimodal imaging to further evaluate retinal microvasculature, retinal sensitivity and photoreceptor arrangement.

## Case report

### Clinical evaluation

**Fig. 1:**
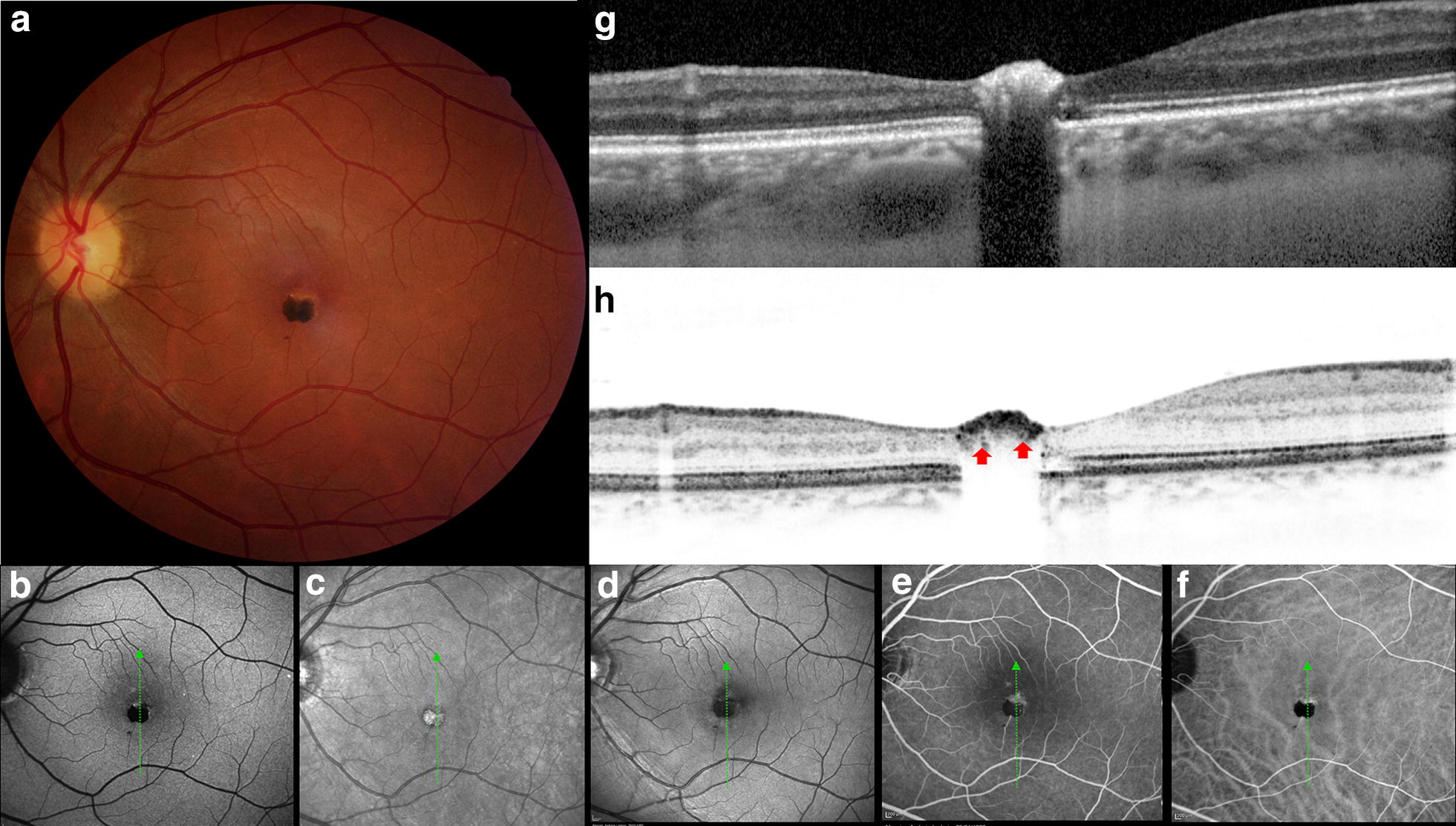
Multimodal assessment. **a** Color fundus photograph of hamartoma mass, 0.6 mm (H) × 0.5 mm (V) in diameter with yellowish superior border. **b** Autofluorescence filter signal blocked by the pigmented lesion. **c** Near-Infrared hyperreflectivity on the lesion. **d** Red free signal blocked by the pigment. **e** FA revealing hyperfluorescence feeder vessel and superior margin in the artery-venous phase. **f** Indocianine Green angiography picture depicts lesser cyanescence on superior boundary. **g** EDI-SBOCT demonstrating intrinsic hyperreflectivity in almost all foveal layers and shadowing posterior to it, involving the choriocapillaris. **h** SBOCT revealing detailed rounded hyperreflective vessels (red arrow) below the hyperreflective mass

**Fig. 2:**
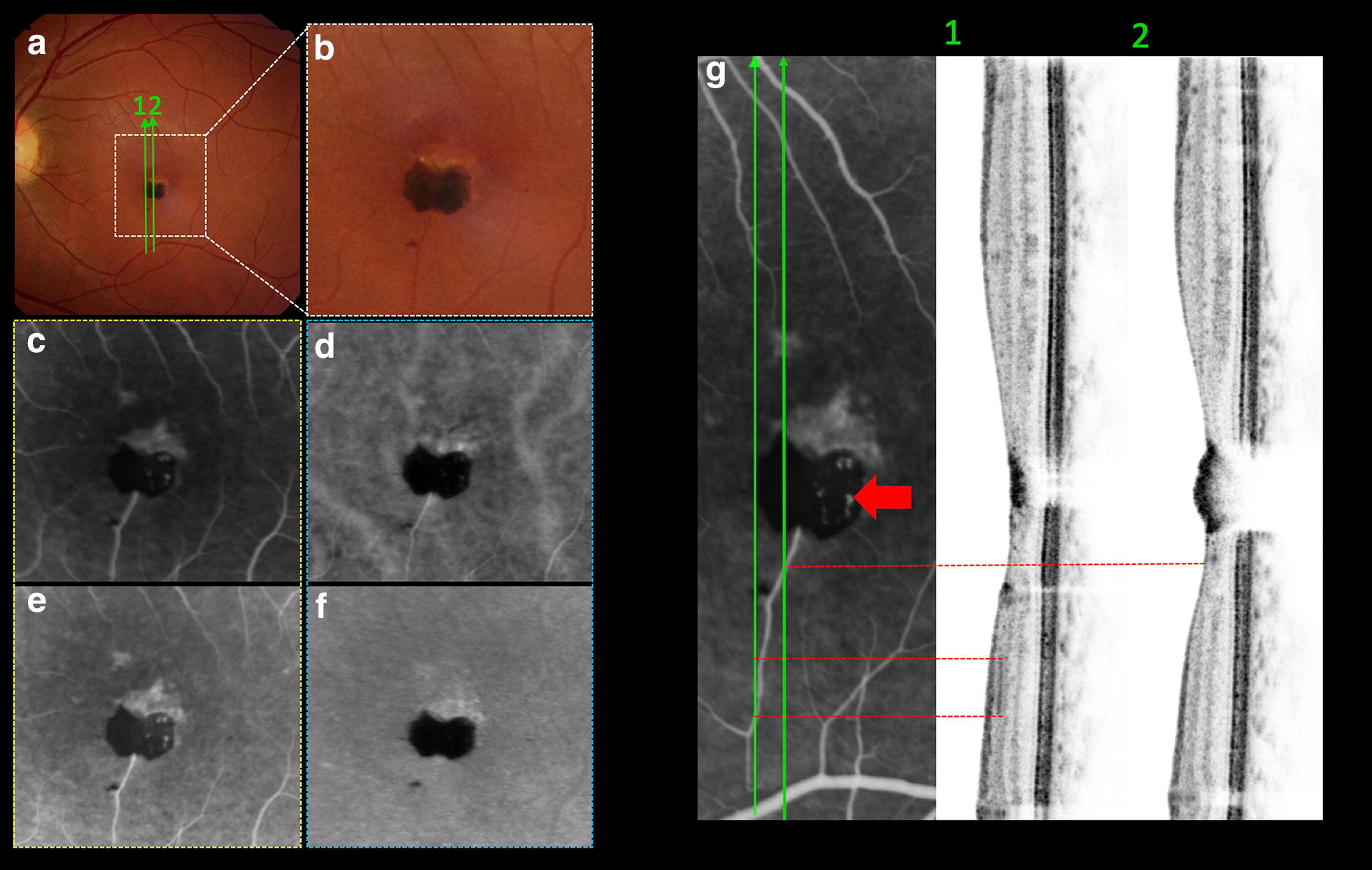
Comprehensive retinal feeding vessel evaluation. **a** Color fundus photograph showing feeder vessel sectioning (green arrow 1 and 2). **g1**, **g2**, SBOCT crossing the hyperfluorescent feeder vessel (red dashed lines). Intrinsic mild ring-shaped fluorescence is well noted (**g**, red arrow). **b** High view of feeder vessel toward to inferior pigmented lesion. **c**, **d**, early fluorescein and indocyanine phase respectively. **e**, **f**, late fluorescein and indocyanine phase respectively

A 35-year-old white woman was found on routine evaluation to have a solitary, circumscribed, nodular, and heavily pigmented retinal lesion in her left eye (OS). Visual acuity was 20/20 in the right eye (OD) and 20/25 in OS. The anterior segment and intraocular pressure were normal in both eyes, as was fundus examination of the OD. Fundus examination OS (Fig. 1a) showed a juxtafoveolar pigmented, circumscribed lesion measuring 0.6 mm in horizontal basal diameter and 0.5 mm in vertical basal diameter. There was a minimally dilated feeding retinal arteriole across the lesion splitting the mass into a bi-lobulated “butterfly” appearance (Fig. 2b). There was no macular edema, exudation, hemorrhage, traction or subretinal fluid. The superior-peripheral portion of the lesion was less pigmented, and characterized by yellowish border. These findings were consistent with CSHRPE.

### Heidelberg HRA-multimodal assessment

Near-infrared reflectance imaging (Fig. 1c) demonstrated intrinsic hyperreflectivity, whereas short-wavelength autofluorescence (Fig. 1b) and red-free filter photography (Fig. 1d) revealed blocked signal by the lesion. Fluorescein angiography (FA) revealed mild ring-shaped fluorescence (Fig. 2g, red arrow) of the lesion in the arteriovenous phase that persisted without leakage into the late phase. Also, the FA showed early fluorescence (Fig. 2c) and late stained fluorescence (Fig. 2e) in the superior border lesion corresponding to the yellowish lesion. There was an outer retinal defect (Fig. 1g) on vertical section of EDI-SBOCT and of SBOCT (Figs. 1h, 5f on asterisk). Indocyanine green not revealed intralesional cyanescence, but only weak cyanescence on superior boundary (Fig. 2d–f). The mass appeared as a highly reflective lesion with deep shadowing by SBOCT (Fig. 1g, h). Interestingly rounded reflective vessels on SBOCT imaging (Fig. 1h, red arrow) was correlated to the Fig. 2g (red arrow). The feeder vessel was cross-sectioned by SBOCT scans on Fig. 2g1, g2 (red dashed line) in the inner retina layer.

### En face and OCT angiography imaging

**Fig. 3:**
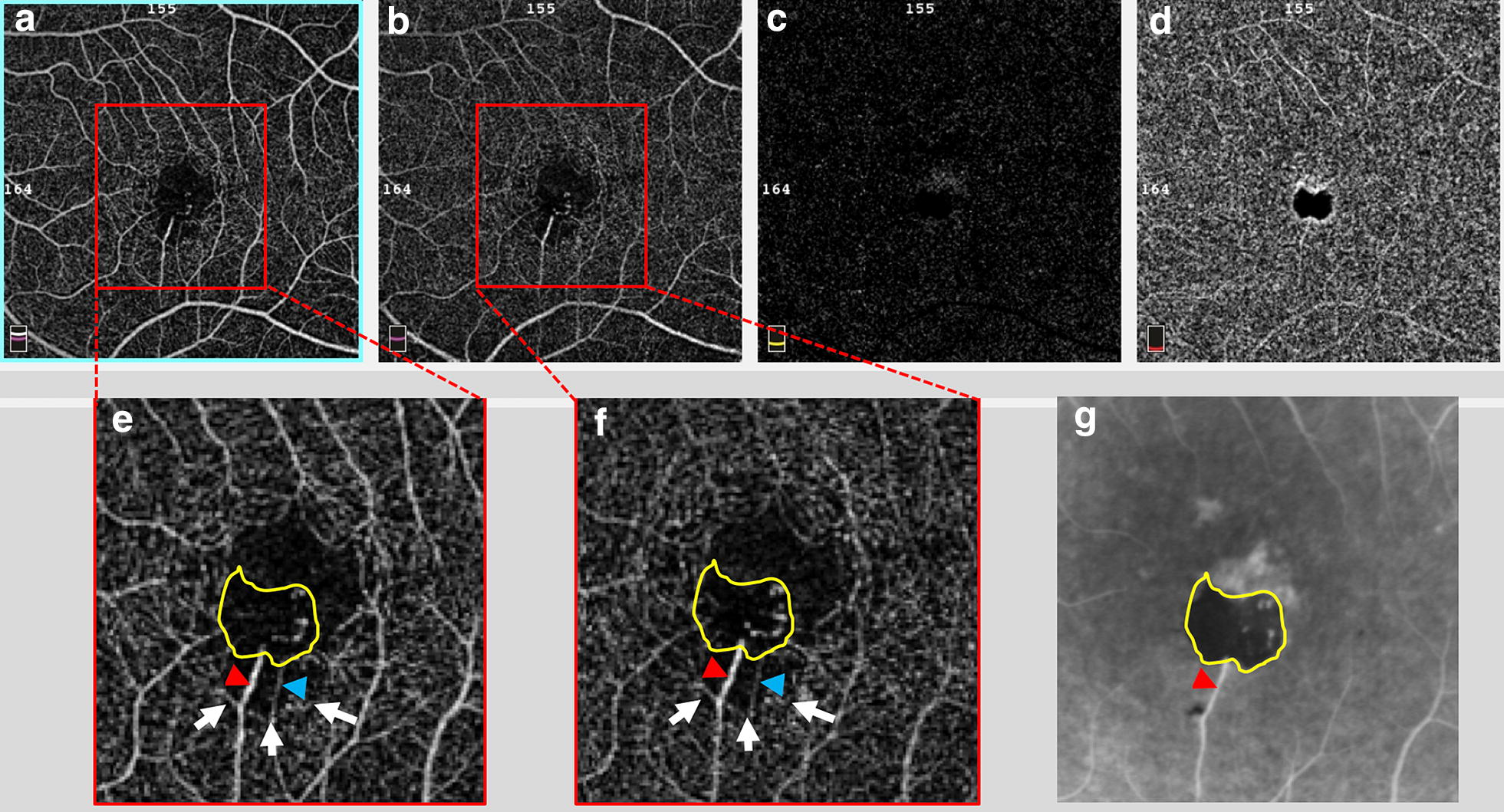
OCT-angiography imaging and fluorescein angiography correlation. OCTA pictures from **a** superficial capillary layer, **b** deep capillary layer, **c** outer nuclear layer and **d** choriocapillaris. **e**, **f** Magnified images (red dashed) focusing increased FAZ (white arrow) and presence feeder arteriole vessel (red head-arrow) paired to drainage venule vessel (blue head-arrow). **g** Related FA area (yellow lined demarcation) showing hyperfluorescent intralesional radial vascularization and stained feeder vessel (red head-arrow)

**Fig. 4:**
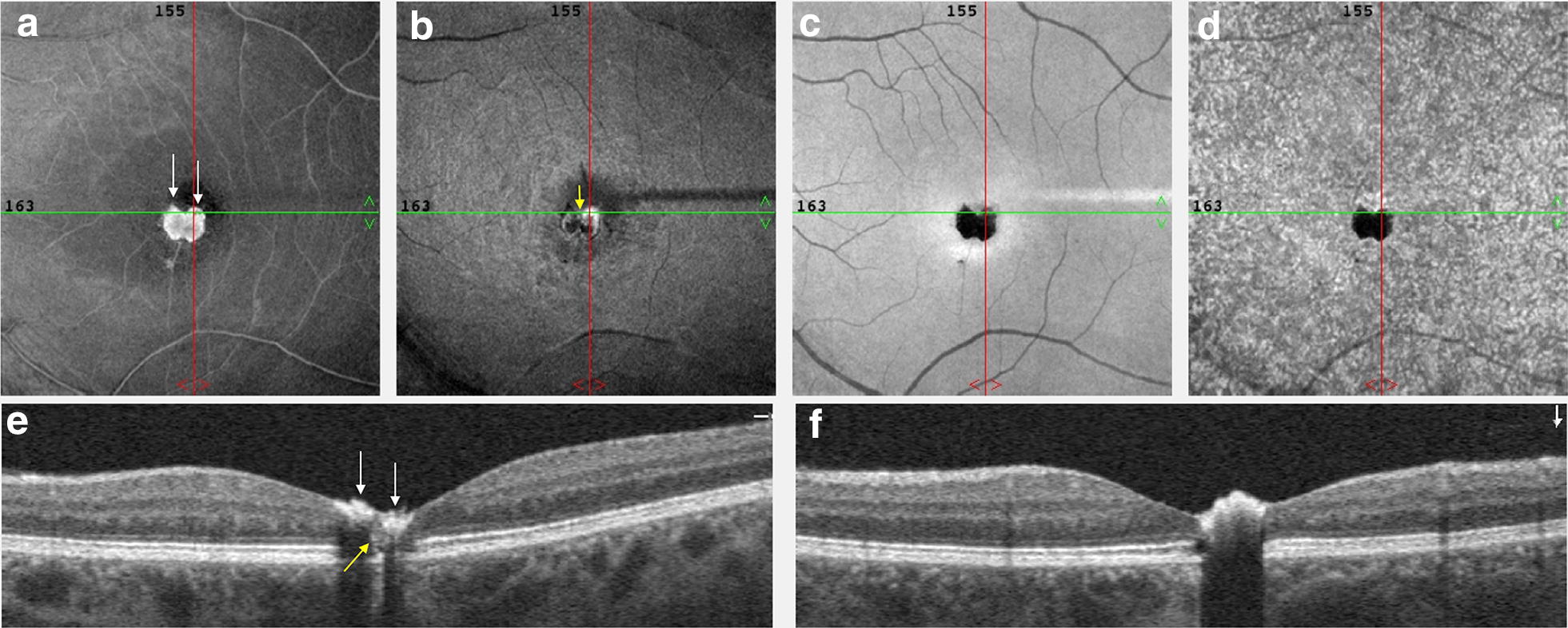
En face OCT imaging. **a** ILM en face image showing hyper signal on pigmented lesion (white arrows). **b** IPL en face level showing deeper bright signal temporally. **c**, **d** RPE and choriocapillary en face level respectively, demonstrating the dark artefact secondary to optical transmission blocking by the hamartoma. **e** Horizontal OCT B-scan section showing the hiperreflective tissue on the superficial layers. A very small portion of the outer retina reflectivity may be seen between the two islands of the hamartomatous tissue (yellow arrow). **f** Vertical OCT B-scan section revealing hyperreflective pigmented portion and full thickness shadowing

The images were captured with the standard macula protocol with a resolution of 2 mm × 2 mm from the Avanti (Optovue) system [8]. Hereby, the upper border of the superficial vascular layer was defined as 3 μm below the internal limiting membrane (ILM) and the lower border as 15 μm below the inner plexiform layer (IPL). Subsequently, the OCT-A depicted superficial (Fig. 3a) and deep (Fig. 3b) capillary layer which were zoomed (Fig. 3e, f) and manually correlated to fluorescein angiography (Fig. 3g). Remarkably capillary path was easily detected on OCT-A in comparison to arteriovenous transit on FA. Besides, the feeder vessel (Fig. 3e, f, red arrowhead) was evident a venule-drainage capillary (Fig. 3e, f, blue arrowhead) mainly at superficial capillary level (Fig. 3a, e). A barely visible venule drainage capillary was solely detectable on early vascular FA-transit (Fig. 2c). In addition, the foveal avascular zone (FAZ) was more easily estimated on OCT-A, and was found to be increased FAZ inferiorly to the pigmented lesion (Fig. 3e, f, white arrows). The outer nuclear layer (Fig. 3c) and choriocapillaris (Fig. 3d) layer segmentation showed an artefact dark image from the pigmented lesion, blocking the under visualization. Vertical and horizontal SBOCT sections (Fig. 4e, f) and en face imaging (Fig. 4a–d) were correlated. The ILM en face image showed bright signal on the pigmented lesion (Fig. 4a) that correlated to the hyperreflective SBOCT within retinal inner layer (Fig. 4e, white arrow). In the IPL level (Fig. 4b) the en face image showed deeper bright signal temporally. Additionally, in the interior of the SBOCT shadowing region there was less reflectivity in the outer retina with external limiting membrane (ELM) layer and ellipsoid band absence and that could be related to the deep capillary shunt (Fig. 4e, yellow arrow). The RPE (Fig. 4c) and choriocapillaris (Fig. 4d) en face image demonstrating dark artefact, blocking the optical transmission.

### Microperimetry

**Fig. 5:**
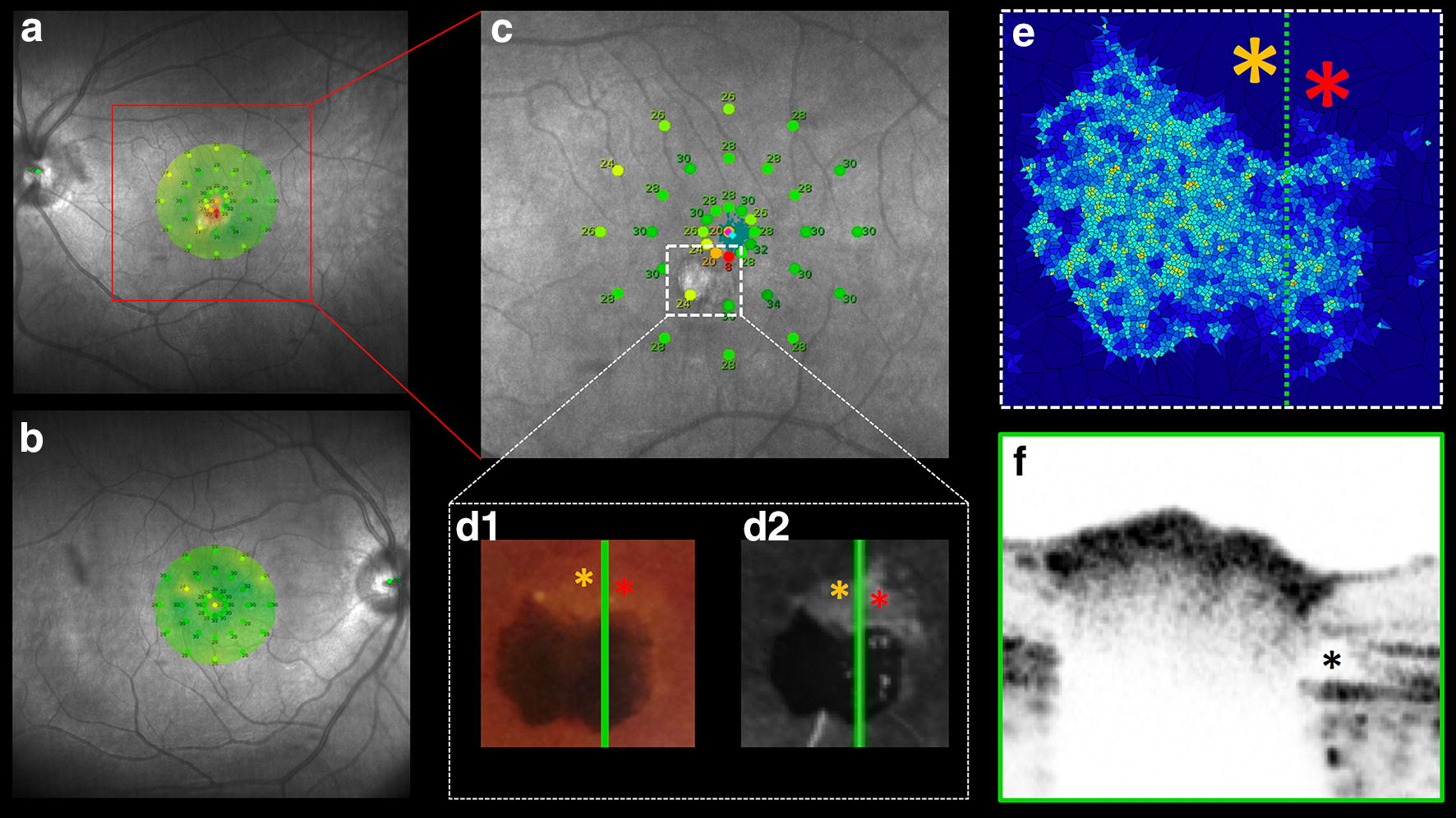
Sensitivity map and imaging correlating evaluation. **a**, **b** microperimetry from OS and OD respectively. **c** Apparent reduced retinal function near to the hamartoma (white dashed square). **d** Correlated hyposensitive area (orange and red asterisks) on yellowish color image as well as the fluorescent margin. **e** Related AO cell density hamartoma showing lack photoreceptor count on hyposensitive area (orange and red asterisks). **f** SBOCT evidencing absence of ellipsoid band and RPE interdigitations in the outer nuclear layer defected

Microperimetry showed an abnormal macular integrity index of 72.9 OS and normal macular integrity index of 9.5 OD, (0–40 indicates normal; 41–60, suspicious; and 61–100, abnormal) [9]. The mean threshold was 27.3 and 28.2 dB, respectively (> 25 to 36 dB indicates normal; 24–25 dB, suspicious; and < 24 dB, abnormal) [9]. A sensitivity map of OS (Fig. 5a, c) suggested reduced retinal function near the hamartoma area, conversely to the regular retinal function from sensitivity map of OD (Fig. 5b). Corresponding hyposensitivity areas (yellow and red asterisks) on yellowish color fundus (Fig. 5d1), hyperfluorescent on FA (Fig. 5d2), cell density absence (Fig. 5e) and SBOCT (Fig. 5f, black asterisk) were related.

### AO imaging and AO system analysis

**Fig. 6:**
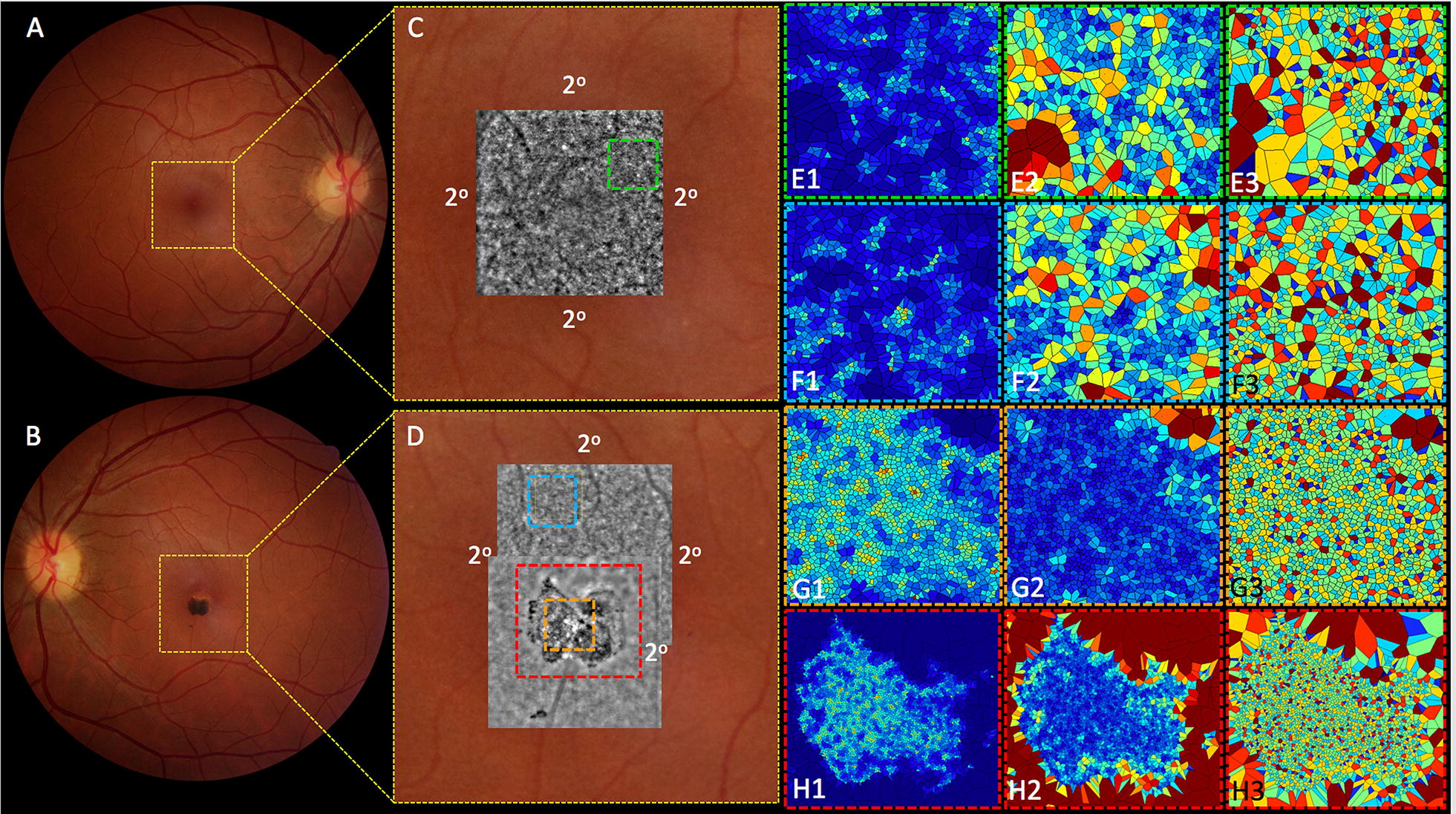
Adaptive optics imaging and AO system analysis. **A**, **B** color image focusing foveal zone (yellow dashed square). **C**, **D** showing adaptive optics en face image (1.2 mm × 1.2 mm) inward to 2-degree foveal center. **E** green dashed square of OD and **F** blue dashed square of OS uninvolved juxta-foveolar retina depicts 300 × 300 µm samples analysis for the cone cell density (parts **E1** and **F1**), intercellular spacing (parts **E2** and **F2**), and Voronoi polygon (parts **E3** and **F3**). **G** orange dashed square of hamartoma region depicts 300 × 300 µm sample analysis for cone cell density (**G1**), intercellular spacing (**G2**), and Voronoi polygon (**G3**). (**H**_**1**–**3**_); entire hamartoma analysis (red dashed square)

The en face AO imaging inward to the macular 2-center degree (2°) demonstrated an irregular reflective cell mosaic that became discontinuous with enlarged hyperreflective and hyporeflective polygonal shapes at the site of the lesion (Fig. 6D, red dashed square). High cone cell density was detected despite the tumor presence (Fig. 6D, orange dashed square). The unaffected matched superior nasal retina in both eyes within 2° on juxtafoveolar area revealed cone photoreceptors as continuous bright hyperreflective dots (Fig. 6C, green dashed square and D, blue dashed square). Representative 300 × 300 µm sampling windows from uninvolved (Fig. 6C, green dashed square and D, blue dashed area) and hamartoma (Fig. 6D, orange dashed square) areas were matched to spatial distribution inside 2° centered area and analyzed for standardized RTX 1 measurements which included intercellular spacing (normal range 15–20 µm), local cell density (normal range 20,000–30,000 cones/mm^2^), and number of neighboring cells (standardized value: approximately 6) using the AO software images to create color scale graphic representation [10]. Measurements obtained in unaffected superior-nasal area of OD, OS and hamartoma area (OS, inferiorly) revealed mean cone densities of 7355 (Fig. 6E1), 7175 (Fig. 6F1), and 16,375 (Fig. 6G1) cones/mm^2^, respectively. Intercellular spacing was 11.98 (Fig. 6E2), 12.46 (Fig. 6F2) and 8.52 (Fig. 6G2) µm in the uninvolved superior-nasal area of OD, OS and hamartoma area respectively. Proportions of 6-sided Voronoi polygons were 33.8% (Fig. 6E3), 33.5% (Fig. 6F3) and 38.6% (Fig. 6G3) in the uninvolved superior-nasal area of OD, OS and hamartoma area respectively.

## Discussion

Tumors of the RPE are uncommon. They are classified into diagnostic groups including CSHRPE, congenital hypertrophy of the RPE, combined hamartoma of the retina and RPE, congenital albinotic and amelanotic spots of the RPE, torpedo maculopathy, and congenital RPE dysgenesis [1]. It has been theorized to be a congenital proliferation of RPE cells with benign cytological features that aberrantly migrated to involve all layers of the neurosensory retina [2, 3]. Accordingly, b-scan OCT images from the present study demonstrate deep architectural modifications under the pigmented part of the lesion (Figs. 1G, 4E).

In agreement to Gass [3] description of intrinsic vasculature as an element to characterize CSHRPE, the present report also demonstrated an exclusive vascular circuit distinguishing from other OCTA reports [6, 7], as well as an evident patent feeder vessel (Figs. 2, 3) as published by Shields and colleagues [1] in 100% of their cases.

According to the literature, surrounding mild retinal traction can be associated to CSHRPE in 80% cases [1]. In one surgical case, vitreomacular traction (VMT) and secondary retinal thickening associated with CSHRPE lead to surgical repair and postoperative histopathological specimen analysis demonstrated a nodular proliferation of hyperplastic RPE cells with attached gliotic retina and ILM, similar to current histological studies from VMT and epiretinal membrane (ERM) surgeries [11, 12]. This case [12] involved an elderly patient with important vitreo-macular traction dragging the inner retina into a pre-retinal macular fibrosis formation. Subsequently, predictable large presence of fibroblastic tissue and less significant angiomatous component was confirmed on small specimen samples. The postoperative OCT after retinal surgical revealed only partial-thickness retinal defect with preservation of outer nuclear layer (ONL), including discontinuous ellipsoid band and RPE atrophy. Differently, our case reported herein demonstrated no VMT, but also showed partial-thickness retina involvement as mentioned above.

For the first time in the literature according to a Medline search, the present report confirms the presence of cones inner/outer segments in a CSHRPE using adaptive optics analysis (Fig. 6D-orange dashed square and Fig. 6G1, cone cell density). In this regard, two representative juxta-foveolar retina images were matched for comparison (Fig. 6C-green dashed square and Fig. 6D-blue dashed square). On the lesion area, there was a center portion with a very good cell density around 16.000 cells (normal range 20.000–30.000) surrounded by a region of complete absence of cones that corresponded to areas of retina and RPE atrophy. Despite below the normal range values, the island of good cell density on the hamartoma region had higher density than the matched unaffected retina. As expected, inward to two (2°) centered degree the juxta-foveolar unaffected areas from both eyes showed reduced cone-cells detection (Fig. 6E1, F1); not obligatory meaning photoreceptor cell death but reduced regular capability of software cell detection due to excessive packing of cones inner/outer segments in this 2° region [13]. In other words, our report shows that there are cones in good density on an CSHRPE, and maybe the unpacking effect of the lesion on photoreceptors architecture may facilitate cone inner/outer segments detection, leading to higher values of cell density when compared to normal unaffected retina.

In an effort to explore the potential retinal function at the site of this pigmented lesion and surrounding margin, we used microperimetry to determinate the sensitivity response (Fig. 5a, b). The OS-sensitivity map detected regular sensitivity response over the pigmented tumor as corroborated by the photoreceptor density findings using AO. Compared to the OD, the OS-sensitivity map detected a focal reduced retinal sensitivity among corresponding areas (yellow and red asterisks) of yellowish RPE atrophy (Fig. 5d1) presented as transmission FA-hyperfluorescence (Fig. 5d2), In this area, there is also SBOCT-ellipsoid and outer segment hyper reflective layers defects (Fig. 5f, black asterisk) and reduced photoreceptor density on AO analysis (Fig. 5e). In fact, external retinal layer disruption has been reported [14] and Hypothetical explanations include RPE rearrangement and anomalous inner retinal allocation sustained by feeder vessel nourishment.

Strengths of this study include the systematic qualitative multiple correlated imaging assessment from advanced technology using multimodal devices to better understand the architectural changes of CSHRPE. Limitations of this study include the ones usually related to the case report nature of the manuscript. Small number with limited variability of data. Limitations of the imaging techniques such as cone density measurements as commented above.

In summary, OCT-A, adaptive optics, and microperimetry findings provide novel data that could predict functional visual outcome of this tumor. Future research multimodal imaging of this rare condition is warranted to better understand the pathophysiology and visual impact of this condition.

## Abbreviations

RPE: retinal pigment epithelium
EDI-SBOCT: enhanced-depth imaging spectralis B-scan optical coherence tomography
OCT-A: optical coherence tomography angiography
CSHRPE: congenital simple hamartoma of the retinal pigment epithelium
FA: fluorescein angiography
ILM: internal limiting membrane
FAZ: foveal avascular zone
IPL: inner plexiform layer
ELM: external limiting membrane
VMT: vitreomacular traction
ERM: epiretinal membrane
ONL: outer nuclear layer
VEGF: vascular endothelial growth factor
